# Delegates from Massachusetts to the National Med. Convention

**Published:** 1846-12

**Authors:** 


*Delegates from Massachusetts to the National Med. Convention.*—We regret to perceive by an editorial article in a late No. of the Boston Med. and Surg. Journal, that there is some uncertainty as to the election of delegates to represent the Mass. Med. Society at the next National Convention. What! the old Bay State backward in a movement, the aims of which are *reform* and *progress!* We cannot think that the profession of the State will sustain this course, if determined on by the counsellors of the society. From personal acquaintance with the views of a considerable portion of the profession in that state, we know that great interest, out of Boston at least, was felt in the last Convention; and it was agitated in some counties as to the propriety of sending delegates independently of the State Society. We hope this will be done. Delegates chosen by County Societies obviously afford the fullest and fairest representation of the body of the profession. We presume the N. Y. State Society will not attempt again to cut off representation from the County Societies of this State. It appears that at the last annual meeting of the Mass. Med. Society, no action was taken on the subject of sending delegates; hence, the question, in so far as that Society is concerned, will devolve upon what are called the Counsellors, composed of a few members residing, for the most part, at Boston. Should they decide not to send delegates, (which we cannot think will the case,) we sincerely hope the County societies throughout the State will not fail to send each its representatives. Indeed, if the State society be represented we hope the local associations will also have their delegates. As a native of the Bay State, we should, for one, feel mortified at any indisposition in that state to co-operate in an enterprise in which the profession of the whole country have so deep an interest.

